# Liposomal amphotericin B and complement activation-related pseudoallergy (CARPA)

**DOI:** 10.1128/aac.01692-24

**Published:** 2025-01-30

**Authors:** George R. Thompson, Gelina M. Sani, Monica A. Donnelley, Jaimie K. Figueroa, Ryan Ciuffetelli, Kate Trigg, Juan Arredondo, Alan Koff, Marie Nearing, Ikaika C. Loque, Derek J. Bays, Satya Dandekar

**Affiliations:** 1 Department of Internal Medicine, Division of Infectious Diseases, University of California-Davis Medical Center 21772 , Sacramento, California, USA; 2 Department of Medical Microbiology and Immunology, University of California-Davis 272916 , Davis, California, USA; 3 Department of Pharmacy, University of California Davis Medical Center 21772 , Sacramento, California, USA; University of Iowa, Iowa City, Iowa, USA

**Keywords:** amphotericin B, liposome, adverse drug reaction, hypertension

## Abstract

Infusion reactions (tachycardia, hypertension, fever, etc.) associated with liposomal amphotericin B are common. Animal models have found complement activation responsible, yet the pathophysiology has not been evaluated in human patients. We performed a prospective observational study and found complement activation-related pseudoallergy (CARPA) responsible in those with infusion reactions.

## INTRODUCTION

Advances in the prophylaxis and treatment of invasive fungal diseases have reduced associated patient morbidity and mortality. The triazole and echinocandin antifungals are recommended as first-line therapeutic agents in the treatment of invasive aspergillosis and candidiasis, respectively; however, amphotericin B (AMB) formulations are still required during the treatment of patients who are refractory or intolerant to treatment, or with infections resistant to other antifungal classes.

Amphotericin B deoxycholate (AMB-d) was approved in 1959 yet an unfavorable side effect profile subsequently prompted the development of alternative formulations. Amphotericin B was thereafter reformulated with lipids to replace the deoxycholate component, and these formulations possess significantly reduced toxicity profiles (1). Liposomal amphotericin B (L-AMB) consists of spherical unilamellar vesicles approximately 60–70 nm in size with AMB molecules dispersed throughout the surface of the lipid bilayer (2).

Liposomal medications carry the risk of hypersensitivity and/or infusion reactions, and L-AMB infusion reactions have been documented to occur in 6%–9% of patients (3), although real-world studies have shown rates closer to 20% (4). Animal models have found liposome-associated reactions are non-IgE-mediated and termed pseudoallergy, with activation of the complement system as the underlying cause, termed complement activation-related pseudoallergy (CARPA), and associated symptoms include hemodynamic changes, flushing, rash, urticaria, chest and back pain, dyspnea, and/or fever (5). Infusion reactions observed in patients may be severe and necessitate drug cessation, although most are minor and transient. Evidence of CARPA secondary to L-AMB has not been definitively demonstrated in a human cohort of patients and we sought to evaluate the immunologic effects of L-AMB infusion.

We performed a prospective observational study of patients receiving L-AMB therapy. Patients were identified by notification from a UC-Davis pharmacist following a prescription for amphotericin B in treatment-naïve patients. All eligible patients were approached for potential participation. Demographic data, comorbidities, concurrent medications, treatment indication(s), vital signs, symptoms, and laboratory values were collected. The institutional review board of the University of California-Davis Medical Center approved this study.

Following informed consent, peripheral blood samples were collected immediately prior to L-AMB infusion (time 0) to serve as baseline values, 5 minutes into infusion, and 30 minutes following completion of infusion (1 mL at each time point). L-AMB was infused per hospital protocol over a period of 60 minutes. If L-AMB was still required 7 days after their initial infusion, peripheral blood samples were again collected at time 0, 5 minutes, and 30 minutes following infusion to determine if the complement/immunologic responses extinguished with repeated exposure. Pre-treatment with antihistamines or other medications was at the discretion of the primary physician(s) of record. Blood specimens were stored at −80°C. C3a and C5-9 levels were measured by enzyme-linked immunosorbent assay (ELISA) (MicroVue, Quidel Corporation). Cytokine measurements were also obtained by ELISA and included IL-1β (Ebioscience, Thermo Scientific), IFN-γ (Fisher Scientific), TNF-α (Ebioscience, Life Technologies), and IL-6 (Invitrogen, Thermo Scientific). Data are presented as medians and ranges, and statistical analysis was performed by Wilcoxon matched-pairs signed rank test (GraphPad Prism v6.0, La Jolla, CA, USA).

Thirteen patients were prospectively enrolled with a median age of 54.6 years (range 13–70) and included 8 men and 5 women. Patients included were White (53.9%), Hispanic/Latino (23.1%), Asian (15.4%), and Black (7.7%). Indications for L-AMB included empiric antifungal therapy (*n* = 7), mucormycosis (*n* = 3), hyaline mould not otherwise specified (*n* = 1), dematiaceous mould (*n* = 1), and *Cryptococcus neoformans* (*n* = 1). All enrolled patients received intravenous L-AMB at a dose of 5 mg/kg ideal body weight over 60 minutes infusion time as per local institutional practice.

**Fig 1:**
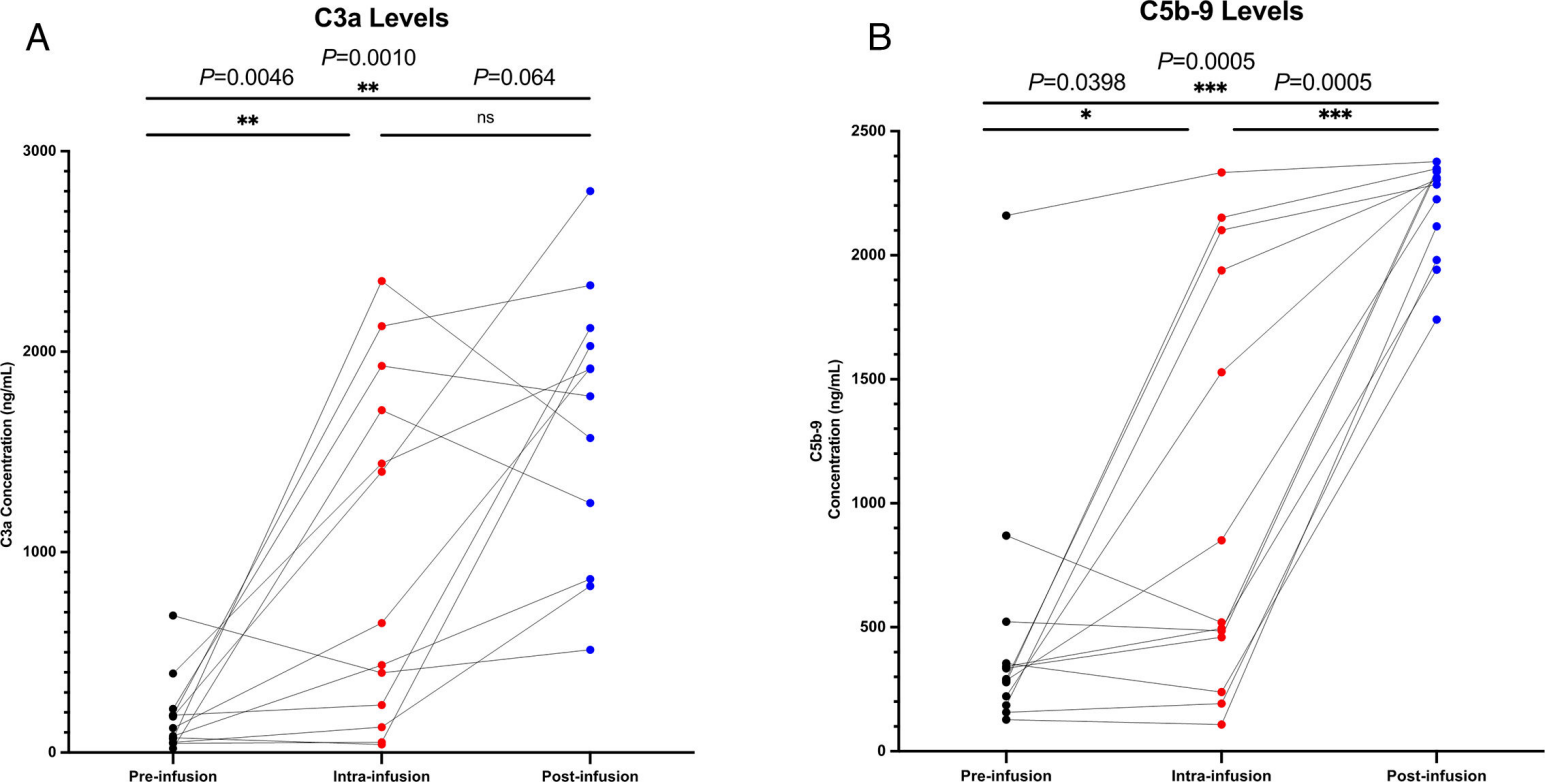
(**A**) Changes in C3a levels during liposomal amphotericin B infusion. (**B**) Changes in C5-9 levels during liposomal amphotericin B infusion.

Significant increases were observed in C3a levels both intra-infusion (5 minutes after treatment initiation) (Δ median 145.3 ng/mL, IQR 208.0 ng/mL, *P* = 0.0046) and post-infusion (30 minutes after completion of infusion) (Δ median 254.9 ng/mL, IQR 64.5 ng/mL, *P* = 0.0010) compared to pre-infusion baseline values (Fig. 1A). C5-9 levels also were observed to significantly increase at intra-infusion (Δ median 152.4 ng/mL, IQR 1,327.6 ng/mL, *P* = 0.0398) and post-infusion (Δ median 1,939.0 ng/mL, IQR 403.3 ng/mL, *P* = 0.0005) time points compared to baseline (Fig. 1B) although interpatient variability is evident. Elevations in complement levels noted during the intra-infusion period continued to increase through the post-infusion period for both C3a (*P* = 0.064) and C5-9 (*P* = 0.0005) suggesting a continued response over the course of infusion.

Prospectively observed infusion reactions were mild and occurred in only four patients (tachycardia, fever [2] [>38°C], nausea, and flushing). Baseline, intra-infusion, and post-infusion C3a and C5-9 levels did not significantly differ between the patients who experienced symptoms compared to those who did not. However, comparing the change in complement levels from baseline to intra-infusion in the four symptomatic patients to those who did not develop symptoms found a significant rise in C3a and C5-9 levels (ΔC3a: *P* = 0.0056; ΔC5-9: *P* = 0.0336).

A subset of patients received L-AMB 7 days after their initial dose and underwent repeated testing (*n* = 7). Despite daily therapy, the effects of L-AMB on complement levels still resulted in non-significant increases in C3a and C5-9 from pre- to intra-infusion, however, the effects were dampened in comparison to their initial L-AMB infusion (C3a: *P* = 0.0781; C5-9: *P* = 0.3750)—findings suggesting the development of tolerance to AMB infusion over time during daily therapy.

Stratification of patient samples by pre-treatment with anti-histamines (*n* = 6), acetaminophen (*n* = 8), or other analgesics (*n* = 8) showed no significant differences between groups, although this study was not specifically powered to assess these differences.

Assessment of cytokine values in both intra-infusion and post-infusion did not show significant changes for IL-1β, IFN-γ, TNF-α, or IL-6 compared to baseline, nor in those with symptomatic infusion reactions (Fig. S1). Additionally, there were no statistically significant differences in cytokine measurements stratified by pre-treatment regimens.

Liposomal drugs carry an increased risk for infusion reactions. Liposomal doxorubicin and L-AMB are those most frequently reported as causal in the development of CARPA, although other agents have been described (6). Immunologic recognition of liposomes by the innate immune system is essential as phospholipid vesicles are similar in size and shape to pathogenic organisms (enveloped viruses) and subcellular organelles (6). Rapid activation of the complement system is therefore a crucial aspect of immediate host defense and occurs prior to the development of an adaptive immune response for pathogen recognition and control of invasion. The precipitous development of CARPA following liposome infusion is thus not surprising and represents a conserved immunologic process aimed at protection from acute infection/exposure.

*In vitro* and animal models assessing the development of CARPA for liposome-containing compounds have been developed. *In vitro* studies have shown inter-patient variability in the release of complement using human sera with liposomal doxorubicin, however, L-AMB caused substantial complement activation in all tested samples suggesting significant differences in reactivity between the lipid agents and formulations (5). Other reports have proven this association by inhibition of CARPA development with anti-C5a monoclonal antibody pre-treatment (7).

Several chemical (PEGylation) and structural (size, shape, and net charge) variables impact complement reactivity to liposomes and these differences are responsible for the effects observed with L-AMB. Other lipid-based formulations of amphotericin B (amphotericin B lipid complex) have also been shown to cause CARPA (8), although amphotericin B in the absence of lipid formulation does not contribute to complement reactivity (5).

Animal models exploring lipid-containing amphotericin B formulations have demonstrated the potential for L-AMB to cause CARPA. A porcine model evaluated complement activation and hemodynamic effects administered via bolus L-AMB into the pulmonary artery (5) resulting in a >300% rise in the pulmonary arterial pressure and a ~60% decrease in the systolic blood pressure within 1 minute and cardiopulmonary arrest was observed in one of the exposed animals. A later study, also using a porcine model and the same administration mechanism, showed a dose-dependent effect of L-AMB administration on pulmonary arterial pressure with accompanying mild and transient (<15 minutes) suppression of white blood cell and platelet counts (9). In the porcine studies, complement activation was associated with cardiovascular infusion reactions, however, the bolus infusion of L-AMB directly into the pulmonary artery limits direct extrapolation of this model to findings observed in humans.

Similarly, our cohort also saw complement levels significantly increase following L-AMB infusion. Additionally, complement increases were most pronounced in patients with clinical signs/symptoms consistent with CARPA (fever, tachycardia, etc.) confirming this association. We did not observe any patient exhibiting a severe reaction to AMB and these reactions may need to be studied in future work.

In the subgroup of patients who received L-AMB for 7 days and had additional samples drawn, complement levels still increased following infusion, yet the change in complement levels from baseline was muted in comparison to changes at the time of initial infusion and no infusion reactions were observed at this later time point. These findings suggest complement activation may diminish with continued L-AMB therapy and provide a rationale for attempts to continue treatment when infusion reactions can be lessened with pre-medication and other maneuvers.

The rate of L-AMB infusion and pre-medication with corticosteroids and/or antihistamines (8, 10), directly impacts the likelihood of developing L-AMB-associated CARPA (5). Among some providers, pretreatment with antihistamines and other agents is considered the standard of care prior to AMB infusion and limits our ability to explore this in a real-world environment.

Complement-independent mechanisms of infusion reactions have also been noted with other liposomal agents and amphotericin B deoxycholate. Drug exposure has been associated with increases in numerous cytokines and expression of innate immune markers (IL-1β, IL-6, TNF-α, MCP-1, and MIP-1β) (11). The pro-inflammatory effects of AMB are well documented with AMB-d, however, these effects are less commonly observed with lipid amphotericin B formulations *in vitro* (12–14) and our results from clinical samples obtained during infusion confirm these *in vitro* findings. A prior study did observe increases in TNF-α and IL-6 following infusion with AMB formulations, although L-AMB resulted in the lowest cytokine changes of tested agents (15). Tissue-specific effects may have occurred that may have not been observed with an assessment of only the intravascular compartment.

In summary, we observed a statistically significant increase in C3a and C5-9 levels following L-AMB infusion, and changes in these values were significantly associated with the development of CARPA. The increases in complement were blunted with continued treatment suggesting ongoing therapy may be possible as complement responsiveness diminishes over time. It may also be possible to pre-medicate patients with CARPA from L-AMB, and re-initiate therapy at a lower dose although this should be explored in future studies.

## SUPPLEMENTAL MATERIAL

Figure S1 aac.01692-24-s0001.docx Changes in cytokine levels over time.

ASM does not own the copyrights to Supplemental Material that may be linked to, or accessed through, an article. The authors have granted ASM a non-exclusive, world-wide license to publish the Supplemental Material files. Please contact the corresponding author directly for reuse.
