# Supplementary material for: Liposomal amphotericin B and complement activation-related pseudoallergy (CARPA)
Source: Antimicrob Agents Chemother. 2025 Jan 30;69(3):e01692-24. doi: 10.1128/aac.01692-24 (PMC11881558; doi:10.1128/aac.01692-24)
Supplement: Figure S1 — Changes in cytokine levels over time. [file aac.01692-24-s0001.docx]

**Supplemental Figure 1. Cytokine concentrations pre-infusion, intra-infusion, and 30 minutes post- liposomal amphotericin B infusion.**

ns=not statistically significant; ng=nanograms; mL=milliliters
